# Retraction Note: Potential use of proprotein convertase subtilisin/kexin type 9 (PCSK9) inhibition and prevention method in viral infection

**DOI:** 10.1186/s12934-024-02594-9

**Published:** 2024-11-25

**Authors:** Khursheed Muzammil, Mohammad Hosseini Hooshiar, Shirin Varmazyar, Thabit Moath Omar, Manal Morad Karim, Sadeq Aadi, Shaylan Kalavi, Saman Yasamineh

**Affiliations:** 1 Department of Public Health, College of Applied Medical Sciences, King Khalid University, Khamis Mushait Campus, Abha, KSA Saudi Arabia; 2 Department of Periodontology, School of Dentistry, Terhan University of Medical Sciences, Tehran, Iran; 3 Department of Medicine, Shahroud Islamic azad University of Medical Sciences, Sharoud, Iran; 4 Department of Medical Laboratory Technics, Al-Noor University College, Nineveh, Iraq; 5 Collage of Pharmacy, National University of Science and Technology, Dhi Qar, 64001 Iraq; 6 College of Dentistry, Al-Mustaqbal University, Babylon, 51001 Iraq; 7 Department of Clinical Pharmacy, Faculty of Pharmacy, Islamic Azad University of Medical Sciences, Tehran, Iran; 8 Young Researchers and Elite Club, Tabriz Branch, Islamic Azad University, Tabriz, Iran

**Retraction: Microbial Cell Factories (2024) 23:90** 10.1186/s12934-024-02355-8

The Editor-in-Chief has retracted this article. An investigation by the Publisher has found a number of articles, including this one, which share similar concerns, involving but not limited to, irregularities with respect to submission and authorship. The Editor-in-Chief therefore no longer has confidence in the results and conclusions presented in this article. Mohammad Hosseini Hooshiar does not agree to this retraction. Manal Morad Karim and Saman Yasamineh did not state explicitly whether they agree to this retraction. Khursheed Muzammil, Shirin Varmazyar, Thabit Moath Omar, Sadeq Aadi and Shaylan Kalavi did not respond to correspondence from the Editor about this retraction.

